# Comparison of preoxygenation with a high-flow nasal cannula and a simple mask before intubation during induction of general anesthesia in patients undergoing head and neck surgery: Study protocol clinical trial (SPIRIT Compliant)

**DOI:** 10.1097/MD.0000000000019525

**Published:** 2020-03-20

**Authors:** Jun-Young Jo, Wook-Jong Kim, Seungwoo Ku, Seong-Soo Choi

**Affiliations:** Department of Anesthesiology and Pain Medicine, Asan Medical Center, University of Ulsan College of Medicine, Songpa-gu, Seoul, Republic of Korea.

**Keywords:** high-flow nasal cannula, preoxygenation, simple facemask

## Abstract

**Background:**

To assess the arterial oxygen partial pressure (PaO_2_) at defined time points during preoxygenation and to compare high-flow heated humidified nasal oxygenation with standard preoxygenation using oxygen insufflation via a facemask for at least 5 minutes, before intubation during induction of general anesthesia.

**Methods:**

This randomized, single-blinded, prospective study will be conducted in patients undergoing head and neck surgery. After standard monitoring, the artery catheter at the radial artery or dorsalis pedis artery will be placed and arterial blood gas analysis (ABGA) for baseline values will be performed simultaneously. Each group will be subjected to 1 of 2 preoxygenation methods (high-flow nasal cannula or simple facemask) for 5 minutes, and ABGA will be performed twice. After confirming intubation, we will start mechanical ventilation and check the vital signs and perform the final ABGA.

**Discussion:**

This trial aims to examine the trajectory of PaO_2_ levels during the whole preoxygenation procedure and after intubation. We hypothesize that preoxygenation with the high-flow nasal cannula will be superior to that with the face mask.

**Study registration:**

This trial was registered with the Clinical Trial Registry (NCT03896906; ClinicalTrials.gov).

## 1 Introduction

Preoxygenation before tracheal intubation is usually achieved by delivering oxygen via a facemask before induction of anesthesia. This potentially prolongs the time available for securing the airway before hypoxemia to approximately 6 minutes.^[1–5]^ After the patient loses consciousness, manual ventilation is usually performed using a facemask, until the tracheal intubation is confirmed. This period can be prolonged if tracheal intubation is attempted multiple times. However, facemask ventilation has traditionally been avoided due to the risk of gastric gas insufflation, which leads to increased intragastric pressure, and increases the risk of pulmonary aspiration of the gastric content. Moreover, if difficult mask ventilation is anticipated, facemask ventilation may not be possible at all. Moreover, 6 minutes of hypoxemia without mask ventilation may be insufficient for intubation and may be catastrophic in patients with difficult intubation.

An ideal preoxygenation protocol should extend the apnea period safely during induction of general anesthesia is essential, to prevent life-threatening events. The high-flow nasal cannula (HFNC), such as the OptiFlow System (Thrive, Fisher & Paykel, Auckland, New Zealand), has the ability to deliver warm and humidified oxygen through a specially designed nasal cannula, which enables comfortable oxygen delivery at rates >70 L/min.^[3,6–8]^ Several studies have showed that nasal delivery of humidified oxygen to paralyzed and anesthetized patients maintains oxygenation and achieves acceptable carbon dioxide concentrations.^[1–3,6,9–13]^ The study protocol involves a single-center, single-blinded, prospective, randomized controlled, and superiority trial, to prove the hypothesis that an extended apneic period with preoxygenation via the HFNC could be more beneficial to patients undergoing general anesthesia during induction compared with preoxygenation with a simple mask.

## 2 Methods

### 2.1 Study setting

The study protocol follows the standard protocol items: recommendation for interventional trials (SPIRIT) guidelines for reporting protocol items for randomized controlled trials (RCTs).^[14]^ The process of recruiting and enrolling the study population began in May, 2019 and is currently in progress at Asan Medical Center. Asan Medical Center is a tertiary academic hospital in Korea. The aim of the study is to assess arterial oxygen partial pressure (PaO_2_) at defined time points during preoxygenation and to compare the 2 methods, that is, preoxygenation with high-flow heated and humidified nasal cannula and standard preoxygenation with oxygen insufflation via a facemask. Assessments will be performed at baseline, 2 and 5 minutes, and immediately after intubation for each patient. Outcome assessors include the same anesthesiologist, who cannot be blinded. However, statisticians responsible for the final analysis will be blinded.

### 2.2 Participants

#### 2.2.1 Inclusion criteria

1) Patients with head and neck cancer undergoing resection surgery planned arterial cannulation and invasive arterial blood pressure monitoring and ABGA
2) Patients aged between 19 and 80 years
3) Patients who voluntarily participated in the study
4) Patients with American Society of Anesthesia physical status 1 to 3

#### 2.2.2 Exclusion criteria

1) Patients who did not wish to participate in the study
2) Patients who were unable to give informed consent because of a language barrier
3) Patients with severe respiratory disease
4) Patients with severe cardiovascular or cerebrovascular disease
5) Patients with severe psychiatric disorders
6) Anyone considered inappropriate for participation by the researchers

### 2.3 Assignment of intervention and common procedure

#### 2.3.1 Randomization and blinding

Patients will be randomly assigned to 1 of 2 groups: the M group or the N group. The randomization scheme was generated using a web-based program (http://www.randomization.com), and the sequentially numbered assignments are blinded to which group each participant is assigned until just before surgery. Although the investigator could not proceed blinded due to the nature of the trial, the participants were blinded to the group assigned. An independent outcome assessor will be assigned as an investigator. Unblinding would not be needed until data analysis.

#### 2.3.2 Intervention (preoxygenation phase)

Group N (HFNC): Apply high-flow heated and humidified nasal oxygen with the OptiFlow System at a flow rate of 30 L/min and an inspiratory oxygen fraction (FiO_2_) of 1.0. Gradually increase the oxygen flow to 60 L/min over the course of the first 2 minutes. Ask the patients to not speak during anesthesia induction, to keep the mouth closed, and to breathe with their noses.

Group M (simple facemask): Perform standard preoxygenation by oxygen insufflation via a facemask using the standard anesthesia ventilators (semicircular system) with 100% oxygen flow of 12 L/min. The patients breathe at tidal volume.

#### 2.3.3 Common procedure applied to all subjects

**Figure 1:**
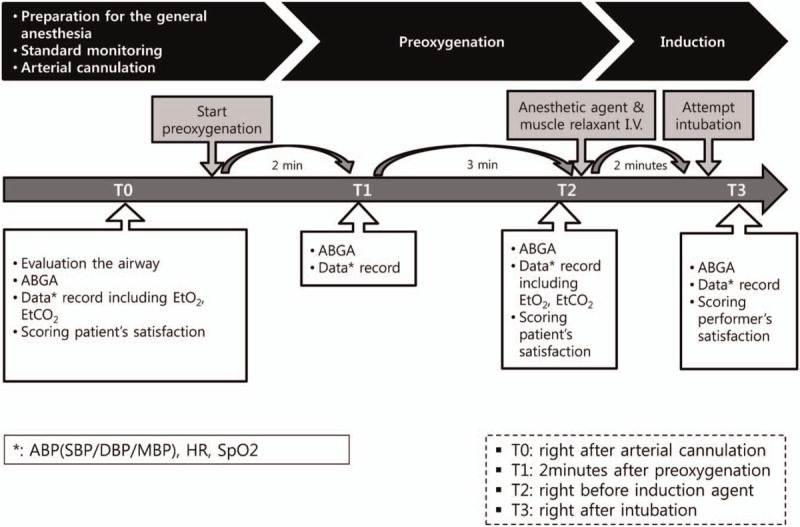
Flow diagram of the study, ∗Data: arterial systolic, diastolic, and mean blood pressure, heart rate, and SpO_2_, T0 = pre-induction baseline, T3 = at second tidal volume, under mechanical ventilation after intubation, ABGA = arterial blood gas analysis, SpO_2_ = oxygen saturation, T1 = 2 minutes after preoxygenation, T2 = 5 minutes after preoxygenation.

Standard monitoring including pulse oximetry (SpO_2_), 3-lead electrocardiogram (EKG), and noninvasive blood pressure will be performed. After local anesthesia, the artery catheter will be inserted into the radial artery or dorsalis pedis artery, with simultaneously baseline ABGA (T0) monitoring. After recording the data from the ABGA and initial vital signs, preoxygenation for a given group will be performed for 5 minutes. Recording of ABGA and the vital signs will be performed at the predefined time points (T1 and T2), before anesthesia injection. Endotracheal intubation will be attempted about 2 minutes after injection of anesthetics and muscle relaxants. After confirming intubation, mechanical ventilation will be started, and the vital signs and last ABGA (T3) will be recorded. The flow diagram of all the study measures is shown in Fig. 1. If the saturation recorded by the pulse oximeter falls below 90% during the procedure, the trial will be stopped immediately. If a patient from group N experiences intolerable discomfort during the course of the trial, the flow rate will be reduced to an acceptable level and recorded. If a patient from group M experiences extreme frustration because of the tight mask, the fitting should be released slightly and record. If appropriate actions are taken for each patient and he/she does not cooperate, he/she will be excluded, even if he/she has consented to participating in the study.

#### 2.3.4 Defined time points

T0: preinduction baseline
T1: 2 minutes after preoxygenation
T2: 5 minutes after preoxygenation
T3: at the second tidal volume with mechanical ventilation after intubation

### 2.4 Outcomes

The primary outcome is PaO_2_ at the predefined time points.

The secondary outcomes include patients’ discomfort, end-tidal oxygen saturation (EtO_2_) at T0 and T3, EtCO_2_ at T0 and T3, other data from ABGA (eg, PaCO_2_, pH, base excess, and HCO_3_), apnea time, SpO_2_, and other vital signs (heart rate, arterial blood pressure).

The tertiary outcomes include the patients’ upper airway condition including the modified Mallampati score, upper lip bite test, thyromental distances and the presence of a mass within the upper airway tract, indication for surgery, seniority of the anesthesiologist, the number of attempts at laryngoscopy, use of any rescue maneuvers, and performers’ (anesthesiologists) satisfaction.^[10,15]^ Apnea time is defined the period from the time of propofol injection till the tracheal tube is secured.

### 2.5 Sample size and recruitment

To determine the sample size for the study, we used previously published data^[9]^ showing that 5 minutes of preoxygenation with HFNC achieved a median of 406 (Q2: 362; Q3: 446) mm Hg, and 5 minutes of facemask preoxygenation achieved a median PaO_2_ of 335 (Q2: 292; Q3: 389) mm Hg. Estimated mean (SD) PaO_2_ achieved with the HFNC and simple mask are 339 (82.3) mm Hg and 405 (71.3) mm Hg, respectively. This yielded a sample size of 23 participants per group, with alpha = 0.05 and power = 0.8.^[16]^ We need 26 participants per group (total 52), after accounting for 10% screen failure. We used a program called G∗Power Version 3.1.7 (Kiel University, Germany) to calculate the sample size.

Patients will be recruited on the day before surgery, once the surgery is planned and the date is scheduled. As soon as sufficient explanation is provided and consent for clinical trials is obtained, they will be assigned to a randomized trial group. After arriving at the operating room for surgery, a monitor for general anesthesia will be started and the clinical trial will be also initiated. There will be no additional visit dates for the participants, as the clinical trial will be terminated as soon as the induction of general anesthesia is completed. Efforts will be made to screen and select the appropriate participants for clinical trials, to recruit participants, and to meet the target sample size.

### 2.6 Data collection management and analysis

After the examiner or the person designated by the examiner receives approval from the institutional review board (IRB), the participants’ data will be collected. To obtain accurate data, the outcome assessors have been given sufficient training, before conducting the trial, and the case report form (CRF) includes pictures and examples for objective assessment of the airway. The collected data should be stored in the original locked location, and access to the data is available only to the responsible investigator and investigators authorized by him.

To compare data between the groups, the chi-square test or Fisher exact test will be used to assess categorical variables. Student *t* test or the Mann–Whitney *U* test will be used to analyze continuous variables, as appropriate. All data manipulations and statistical analyses will be performed using SPSS for Windows, version 21 (IBM Corp, Armonk, NY) and Stata software version 13.1 (StataCorp LP, College Station, TX). Statistical analysis for the missing data will be adjusted with an estimating equation or statistical model in the final analysis. A 2-tailed *P* value of <.05 will be considered to indicate a statistically significant difference.

### 2.7 Monitoring

#### 2.7.1 Adverse events

The investigator should institute appropriate therapeutic and follow-up measures in accordance with good medical practices and should record them in the participant's CRF. The study does not include a data monitoring committee (DMC), but an interim analysis can be made by the investigator responsible, and it will only be made if there is a significant clinical difference between the 2 groups and significant statistical differences could lead to early termination of trial.

#### 2.7.2 Ethics and dissemination

This study was approved by the IRB of the Asan Medical Center, Seoul, Korea (AMC IRB 2019-0275; April 11, 2019). The trial was also registered at Clinical Trial Registry (NCT03896906; April 1, 2019).

This study will be conducted according to the study plan approved by the IRB of our institute and in accordance with the GCP prescribed by the US Food and Drug Administration and ICH. We will do our best to abide by them. Written informed consent will be obtained from all participants by the investigators, before enrollment. The purpose and procedure of the examination will be explained to the participants. Only applicants who have understood the purpose and risk of the examination and completed the consent form can participate in the study. Applicants are aware that they are free to discontinue their participation any time during the study.

The study did not receive any funding, and there are no conflicts of interest. The collected data stored in the locked location can be accessed only by the responsible investigator and the authorized co-investigators. The data will be stored for 3 years after termination of this study, after which it will be thoroughly and completely destroyed. The information obtained through the data analysis will be published in the journal and widely shared.

## 3 Discussion

Preoxygenation with an HFNC during induction of general anesthesia has recently become an appealing option to anesthesiologists.^[3,6,9–12]^ This trial primarily aims to examine the trajectory of PaO_2_ levels during the whole preoxygenation procedure before and after intubation. We expect that the preoxygenation with HFNC will prove superior to the classical method with facemask, during the preinduction period of general anesthesia. To maintain the quality of this study and arrive at a reliable conclusion, the experimental design method and the research results will go through strict quality control. We will use the CONSORT checklist for the experimental design.^[17]^ The main strength of our protocol is that the study population consists of patients with head and neck cancer, who are more likely to have a difficult airway.

## Author contributions

**Conceptualization:** Seong-Soo Choi.

**Data curation:** Wook-Jong Kim, Seungwoo Ku.

**Formal analysis:** Jun-Young Jo, Seong-Soo Choi.

**Methodology:** Jun-Young Jo, Seong-Soo Choi.

**Supervision:** Wook-Jong Kim, Seungwoo Ku.

**Writing – original draft:** Jun-Young Jo.

**Writing – review & editing:** Seong-Soo Choi.

Seong-Soo Choi orcid: 0000-0002-2333-0235.
